# Recombinant Expressed Vector pET32a (+) S Constructed by Ligation Independent Cloning

**DOI:** 10.3390/molecules191016179

**Published:** 2014-10-10

**Authors:** Yu Wang, Guo-Hua Gong, Cheng-Xi Wei, Long Liang, Bin Zhang

**Affiliations:** 1 Medicinal Chemistry and Pharmacology Institute, Inner Mongolia University for the Nationalities, Tongliao 028000, Inner Mongolia, China; E-Mails: shen348@126.com (Y.W.); gongguohua0211@163.com (G.-H.G.); weichengxi1224@163.com (C.-X.W.); 2 State Key Laboratory of Medical Microbiology and Biosafety, Academy of Military Medical Sciences, Biotechnology Institute of Beijing, Beijing 100071, China; 3 Institute of Mongolia and Western Medicinal Treatment, Affiliated Hospital of Inner Mongolia University for the Nationalities, Holin he Street 1742, Tongliao 028000, Inner Mongolia, China

**Keywords:** ligation-independent cloning, vector constructing, prokaryotic gene expression vector

## Abstract

The aim of this work was to develop a new method for constructing vectors, named ligation-independent cloning (LIC) method. We constructed the S label expression vector and recombinant pET32a (+) S-phoN2 by LIC. The recombinant proteins were expressed in *E. coli* at a high level, and then the specificity of the recombinant proteins was identified by western blot. The target band was detected by S monoclonal antibody and Apyrase polyclonal antibodies but not Trx monoclonal antibody and HIS monoclonal antibody. Finally, we obtained protein Apyrase in *E. coli* (BL21), with a protein-only expression S tag. Collectively, our results demonstrated that LIC is effective for the construction of new vectors and recombinant plasmids. Free from the limitations of restriction enzyme sites and with a higher positive rate, LIC processes should find broad applications in molecular biology research.

## 1. Introduction

Generally, the purpose of DNA molecular cloning is the insertion of a particular fragment into a vector to construct a recombinant plasmid. It is widely used in the medicine and biology field. The traditional cloning method involves acquisition of a particular fragment, digestion of a particular fragment and vector connection, transformation and selection.

Traditional cloning methods are widely used in the construction of vectors and recombinant plasmids [1,2]. Some of them have even converted into kits, such as T-A Cloning [3] or Gateway [4,5]. These new technologies have greatly simplified the cloning process, and improved the efficiency of cloning, but some problems still exist. Traditional cloning techniques use restriction enzymes and ligation of DNA *in vitro*, which can be hampered by a lack of appropriate restriction-sites and inefficient enzymatic steps. For example, the restriction enzyme sites of particular fragments and vectors are considered, the particular fragment is treated by a restriction enzyme, and the ligation process needs DNA Ligase [6]. Using traditional cloning methods increases not only the cost of experiments and experiment duration, but also the risk of self-vector connection. In many cases, because of the properties of the original restriction sites of a particular fragment, the experiment has to use some rare restriction. This greatly increases the difficulty and cost of the experiment [7]. A way which is not subject to restriction sites could solve the problem above.

Ligation independent cloning (LIC) has been developed as a new cloning approach which eliminates the use of restriction sites [8]. The principle of LIC is: first of all, the linearized vector and the insert fragment are amplified by PCR with primers containing homologous fragments; second, PCR products are treated with T4 DNA Polymerase (3'-5' exonuclease activity) to obtain the fragment containing 5'-cohesive ends; third, the intermediate is formed by denaturation-renaturation; finally, intermediate is transformed into *Escherichia coli* [9]. The LIC method has advantages over traditional cloning methods in vector and recombinant plasmid construction. LIC eliminates the use of restriction sites and DNA ligease [10]. Using the LIC method to construct vectors and recombinant plasmids not only shortens the experiments, but also reduces the cost of experiments. LIC uses constructed vectors [11], constructed recombinant proteins [12,13] and co-expression proteins [14,15]. LIC is particularly suitable for the construction of new vectors [16] and high molecular weight expression of recombinant proteins [17].

In this work, we report the development of an effective, inexpensive method named the LIC method to construct the expression vector pET32a (+) S and pET32a (+) S-phoN2, which can generate controllable overhangs.

## 2. Results and Discussion

### 2.1. Construction of a pET32a (+) S Expression Vector

**Figure 1:**
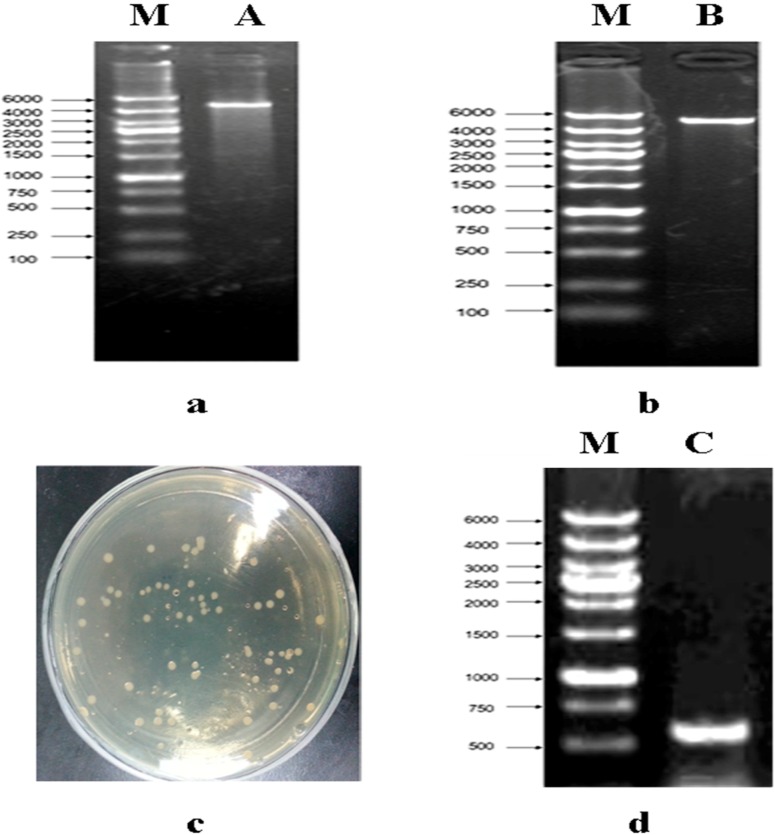
Construction of expressed vector pET32a (+) S. (**a**) line A, MscI digested of vector pET32a (+), the 5900 bp bands were present. Line M, DL6000 Marker; (**b**) line B, PCR products of linear vector pET32a (+), the 5900 bp band was present. Line M, DL6000 Marker; (**c**) the result of colone; (**d**) PCR products of pET32a (+) S, the 604 bp band was present. Line M, DL6000 Marker.

In order to construct the plasmid-only expressed S tag, we used the LIC method to construct pET32a (+) S. Cloning of expression vector pET32a (+) S via LIC was performed as described in Figure 1. After vector pET32a (+) was digested (Figure 1a), it was amplified with a pair of specific primers as described in the Experimental Section. The PCR product was characterized by electrophoresis through 1% agarose gels, and the 5900 bp band appeared as expected with the same size as pET32a (+) (Figure 1b). The transformants were acquired by use of *E. coli* DH5a (Figure 1c), the positive clones were identified by PCR (Figure 1d) and sequenced (Table 1). The result showed the constructed vector pET32a (+) S was not a mutant.

**Table 1:** Sequence analysis of core of pET32a (+) S gene.

CAA GAC CCG TTT AGA GGC CCC AAG GGG TTA TGC TAG TTA TTG CTC AGC GGT GGC AGC AGC CAA CTC AGC TTC CTT TCG GGC TTT GTT AGC AGC CGG ATC TCA GTG GTG GTG GTG GTG GTG CTC GAG TGC GGC CGC AAG CTT GAC GAC GGA GCT CGA ATT CGG ATC CGA TAT CAG CCA TGG CCT TGT CGT CGT CGT CGG TAC CCA GAT CTG GGC TGT CCA TGT GCT GGC GTT CGA ATT TAG CAG CAG CGG TTT CTT TAT GTA TAT CTC CTT CTT AAA GTT AAA CAA AAT TAT TTC TAG AGG GGA ATT GTT ATC CGC TCA CAA TTC CCC TAT AGT GAG TCG TAT TAA TTT CGC GGG ATC GAG ATC GAT CTC GAT CCT CTA CGC CGG ACG CAT CGT GGC CGG CAT CAC CGG CGC CAC AGG TGC GGT TGC TGG CGC CTAA TAT CGC CGA CAT CAC CGA TGG GGA AGA TCG GGC TCG CCA CTT CGG GCT CAT GAG CGC TTG TTT CGG CGT GGG TAT GGT GGC AGG CCC CGT GGC CGG GGG ACT GTT GGG CGC CAT CTC CTT GCA TGC ACC ATT CCT TGC GGC GGC GGT GCT CAA CGG CCT CAA CCT ACT ACT #

Notes: # Length of the sequence was 604 bp; It contained 39–1042 bp of pET32a, where in addition to 294–692 bp.

### 2.2. Construction of a pET32a (+) S-phoN2 Recombinant Plasmid

**Figure 2:**
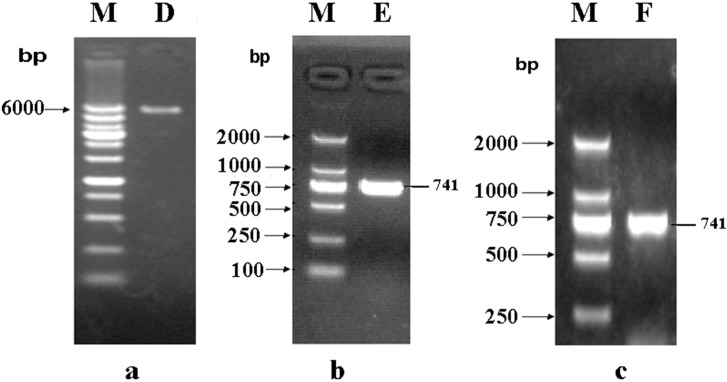
Construction of recombinant plasmid pET32a (+) S-phoN2. (**a**) Line D, the line of linear vector pET32a (+) S (5900 bp). Line M, DL6000 Marker; (**b**) Line E, the line of phoN2 (741 bp). Line M, DL2000 Marker; (**c**) Line F, PCR products of recombinant plasmid pET32a (+) S-phoN2. Line M, DL2000 Marker.

The expression vector pET32a (+) S was amplified with a pair of specific primers as described in the Experimental Section 3.2. The PCR product was characterized by electrophoresis through 1% agarose gels, the 5502 bp band appeared as expected with the same size as pET32a (+) S (Figure 2a).

The quality of the complete genome from *Shigella flexneri* M90T was characterized by electrophoresis through 1% agarose gels. On the basis of the sequence of phoN2 in M90T, gene specific primers were designed. The PCR product was characterized by electrophoresis using 1% agarose gels, and the 741 bp band appeared as expected with the same size as phoN2 (Figure 2b).

The target fragment and linear vector were linked by T4 DNA Polymerase. The transformants were acquired by *E. coli* DH5a and identified by PCR (Figure 2c). The positive clones were sequenced. The result showed the constructed recombinant plasmid pET32a (+) S-phoN2 was not a mutant (Table 2).

**Table 2:** Sequence analysis of Apyrase gene and its encoding amino acids.

ATG	AAA	ACC	AAA	AAC	TTT	CTT	CTT	TTT	TGT	ATT	GCT	ACA	AAT	ATG	ATT	TTT	ATC	CCC	TCA
M	K	T	K	N	F	L	L	F	C	I	A	T	N	M	I	F	I	P	S
GCA	AAT	GCT	CTG	AAG	GCA	GAA	GGT	TTT	CTC		ACT	CAA	CAA	ACT	TCA	CCA	GAC	AGT	TTG
A	N	A	L	K	A	E	G	F	L		T	Q	Q	T	S	P	D	S	L
TCA	ATA	CTT	CCG	CCG	CCT	CCG	GCA	GAG	GAT	TCA	GTA	GTA	TTT	CTG	GCT	GAC	AAA	GCT	CAT
S	I	L	P	P	P	P	A	E	D	S	V	V	F	L	A	D	K	A	H
TAT	GAA	TTC	GGC	CGC	TCG	CTC	CGG	GAT	GCT	AAT	CGT	GTA	CGT	CTC	GCT	AGC	GAA	GAT	GCA
Y	E	F	G	R	S	L	R	D	A	N	R	V	R	L	A	S	E	D	A
TAC	TAC	GAG	AAT	TTT	GGT	CTT	GCA	TTT	TCA	GAT	GCT	TAT	GGC	ATG	GAT	ATT	TCA	AGG	GAA
Y	Y	E	N	F	G	L	A	F	S	D	A	Y	G	M	D	I	S	R	E
AAT	ACC	CCA	ATC	TTA	TAT	CAG	TTG	TTA	ACA	CAA	GTA	CTA	CAG	GAT	AGC	CAT	GAT	TAC	GCC
N	T	P	I	L	Y	Q	L	L	T	Q	V	L	Q	D	S	H	D	Y	A
GTG	CGT	AAC	GCC	AAA	GAA	TAT	TAT	AAA	AGA	GTT	CGT	CCA	TTC	GTT	ATT	TAT	AAA	GAC	GCA
V	R	N	A	K	E	Y	Y	K	R	V	R	P	F	V	I	Y	K	D	A
ACC	TGT	ACA	CCT	GAT	AAA	GAT	GAG	AAA	ATG		GCT	ATC	ACT	GGC	TCT	TAT	CCC	TCT	GGT
T	C	T	P	D	K	D	E	K	M		A	I	T	G	S	Y	P	S	G
CAT	GCA	TCC	TTT	GGT	TGG	GCA	GTA	GCA	CTG	ATA	CTT	GCG	GAG	ATT	AAT	CCT	CAA	CGT	AAA
H	A	S	F	G	W	A	V	A	L	I	L	A	E	I	N	P	Q	R	K
GCG	GAA	ATA	CTT	CGA	CGT	GGA	TAT	GAG	TTT		GGA	GAA	AGT	CGG	GTC	ATC	TGC	GGT	GCG
A	E	I	L	R	R	G	Y	E	F		G	E	S	R	V	I	C	G	A
CAT	TGG	CAA	AGC	GAT	GTA	GAG	GCT	GGG	CGT		TTA	ATG	GGA	GCA	TCG	GTT	GTT	GCA	GTA
H	W	Q	S	D	V	E	A	G	R		L	M	G	A	S	V	V	A	V
CTT	CAT	AAT	ACA	CCT	GAA	TTT	ACC	AAA	AGC		CTT	AGC	GAA	GCC	AAA	AAA	GAG	TTT	GAA
L	H	N	T	P	E	F	T	K	S		L	S	E	A	K	K	E	F	E
				GAA	TTA	AAT	ACT	CCT	ACC	AAT	GAA	CTG	ACC	CCA	TAA				
				E	L	N	T	P	T	N	E	L	T	P	R				

Note: The full-length of the sequence was 741 bp and codes for 247 amino acid residues.

### 2.3. Recombinant Protein Expression

**Figure 3:**
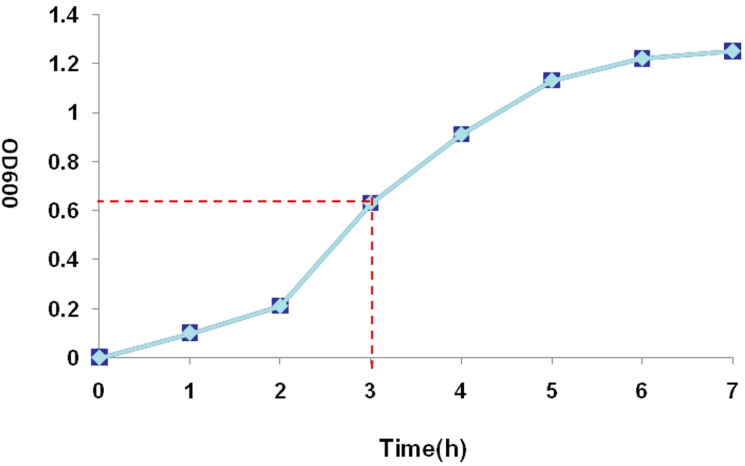
Growth curve of pET32a (+) S-Apyrase/BL 21(DE3). OD600 reached 0.6 at 3 h.

The pET32a (+) S-Apyrase/BL21 was cultivated and OD_600_ was measured every hour. An S-type bacterial growth curve was observed, pET32a (+) S-Apyrase/BL21 was induced at 3 h when they were in the bacterial logarithmic growth phase (Figure 3).

**Figure 4:**
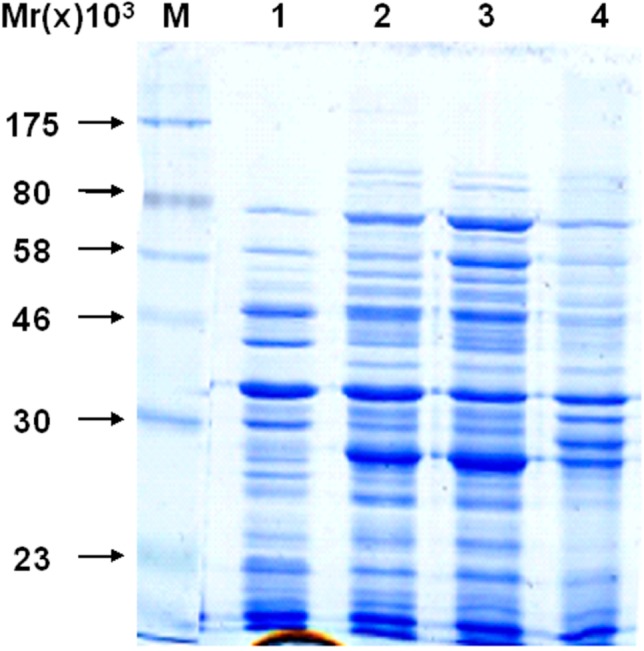
Identification and analysis of the fusion protein by SDS-PAGE. Line M: molecular weight marker: 175 kDa, 80 kDa, 58 kDa, 46 kDa, 30 kDa and 23 kDa; Line 1: whole bacteria of before induce; Line 2: whole bacteria of after induce; Line 3: supernatant of the ultrasound crushing; Line 4: precipitate of the ultrasound crushing.

**Figure 5:**
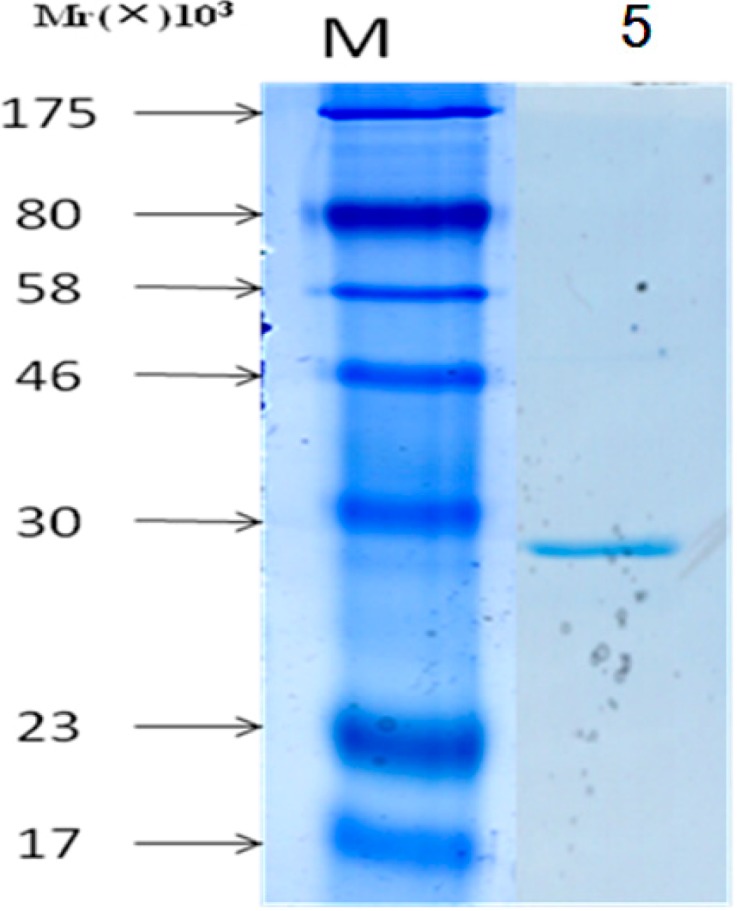
Purified protein Apyrase was identified by SDS-PAGE. Line M: molecular weight marker: 175 kDa, 80 kDa, 58 kDa, 46 kDa, 30 kDa, 23 kDa and 17 kDa; Line A: the purified protein Aprase.

To determine the solubility of recombinant protein, pET32a (+) S-Apyrase/BL21 without induction, whole protein of pET32a (+) S-Apyrase/BL21 after induction, the supernatant of pET32a (+) S-Apyrase/BL21 and the pellet of pET32a (+) S-Apyrase/BL21 were analyzed by SDS-PAGE (Figure 4). The recombinant protein was identified in the induced bacteria but not the control. Comparing the content of ultrasound supernatant and ultrasound pellet, most of the fusion proteins were present in the supernatant, meaning that the fusion protein Apyrase-S was a soluble protein. Lastly, in order to check the activity of recombinant apyrase expression, Apyrase-S protein was purified (Figure 5).

### 2.4. Detecting of the Specificity of Expressing Bacteria

**Figure 6:**
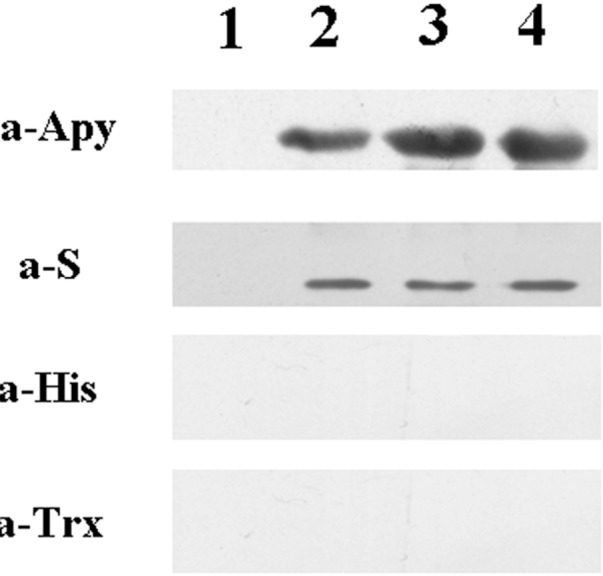
The specificity of expressing bacteria was detected by western blot. Line 1: whole bacteria of before induce; Line 2: whole bacteria of after induce; Line 3: supernatant of the ultrasound crushing; Line 4: precipitate of the ultrasound crushing.

The induced bacteria were lysed, and the specificity of expressing bacteria was analyzed by western blot. The target band was detected by S monoclonal antibody and Apyrase polyclonal antibodies but not Trx monoclonal antibody and HIS monoclonal antibody (Figure 6). This means the expression of fusion protein pET32a (+) S-Apyrase/BL21 only contains S tag. The molecular weight of S tag is only 1.7 kDa, which is too small to affect the structure of the target protein. Using the S tag, we can affinity purify the target protein and combine it with other tags for pull-down experiments. However, pET32a (+) contains two other tags in addition to the S tag. It was very inconvenient to construct a recombinant protein which only expressed S tag, so we constructed pET32a (+) S by LIC, which made the next experiment convenient.

## 3. Experimental Section

### 3.1. Materials

*E. coli* DH5a and *E. coli* BL21(DE3) were purchased from the TIANGEN (Beijing, China); expression vector pET32a (+) was obtained from Novagen (Beijing, China); Antibodies for S, GAPDH, Trx and HIS were purchased from Sigma-Aldrich (Shanghai, China); PrimeSTAR HS DNA Polymerase, T4 DNA Polymerase and IPTG (isopropyl β-d-thiogalactoside) were purchased from the TaKaRa (Dalian, China).

### 3.2. Vector Construction

**Figure 7:**
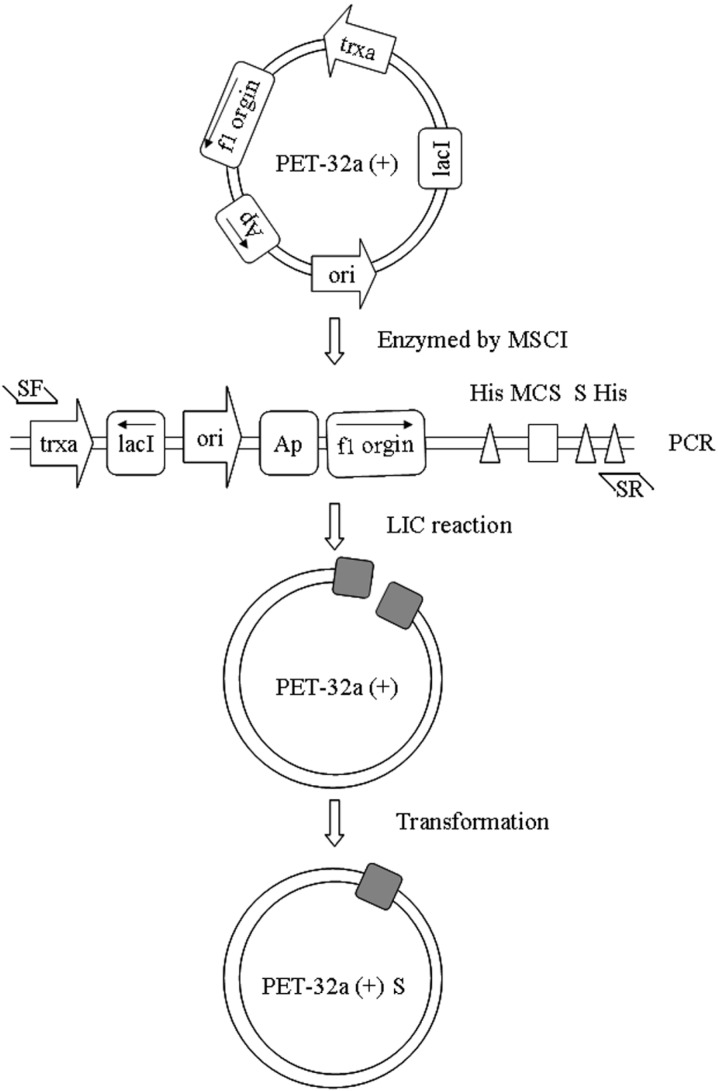
Vector construct.

Construction of the expression vector pET32a (+) S is described in Figure 7. First of all, vector pET32a (+) was treated with MscI. LIC-specific primers were designed as shown below. The primers include homologous fragments. In the process of amplification, the 294–692 bp (Trx tag site) of pET32a (+) was removed:

SF: 5' GGTTTCTTTCATATGTATATCTCCTTCTTAAAGTTAAACA 3'
SR: 5' ATGAAAGAAACCGCTGCTGCTAAATTCG 3'

The PCR was performed in 50 μL reaction mixes using the following cycles: initial denaturation at 98 °C for 30 s; 25 cycles of denaturation at 98 °C for 30 s and extension at 68 °C for 6 min and 30 s; and a final extension at 68 °C for 7 min. PCR products were analyzed by electrophoresis and purified with PCR cleanup kit (Beijing, China).

The cloning reaction was performed by T4 DNA Ploymerase. There was 100 ng DNA model, 10× T4 DNA Polymerase buffers 1 μL, BSA 0.1 μL, T4 DNA Ploymerase 0.1 μL in the best 25 μL restriction system. The linear vector pET32a (+) was made using the following reaction: digestion at 37 °C for 1 min; inactivation at 72 °C for 20 min and annealing at 25 °C. The annealing product was transformed into *E. coli* DH5α, and grown on LB agar plates (ampicillin) at 37 °C for 16 h. LB medium was inoculated with bacterial colonies, the bacterial colonies were grown at 37 °C, 200 rpm for 10–15 h. Then, the bacteria was identified by primers: FSce: 5' CAAGACCCGTTTAGAGGC 3' (39–56 bp); Rsce: 5' AGTAGGTTGAGGCCGTTG 3' (1022–1042 bp).

### 3.3. Construction of pET32a (+) S-Apyrase (Figure 8)

Apyrase was amplified by PCR using AF (5'GCGGATCCATGAAAACCAAAAAC3') and AR (5'CTCGAGTTATGGGGTCAGTTCATTG3') primers. PCR products were analyzed by electrophoresis and purified with a PCR cleanup kit. The linear vector pET32a (+) S was amplified by primers SAF and SAR. The sequences are shown below; the upstream primers and downstream primers included homologous fragments of phoN2:

SAF: 5' GAAAGTTTTTGGTTTTCATGGATCCGATATCAGCCATGG 3'
SAR: 5' CCAATGAACTGACCCCATAACTCGAGCACCACCACCACCA 3'

The cloning reaction was performed by T4 DNA Polymerase. The annealing product was transformed into *E. coli* DH5α, and the bacteria were identified by primers AF and AR.

**Figure 8:**
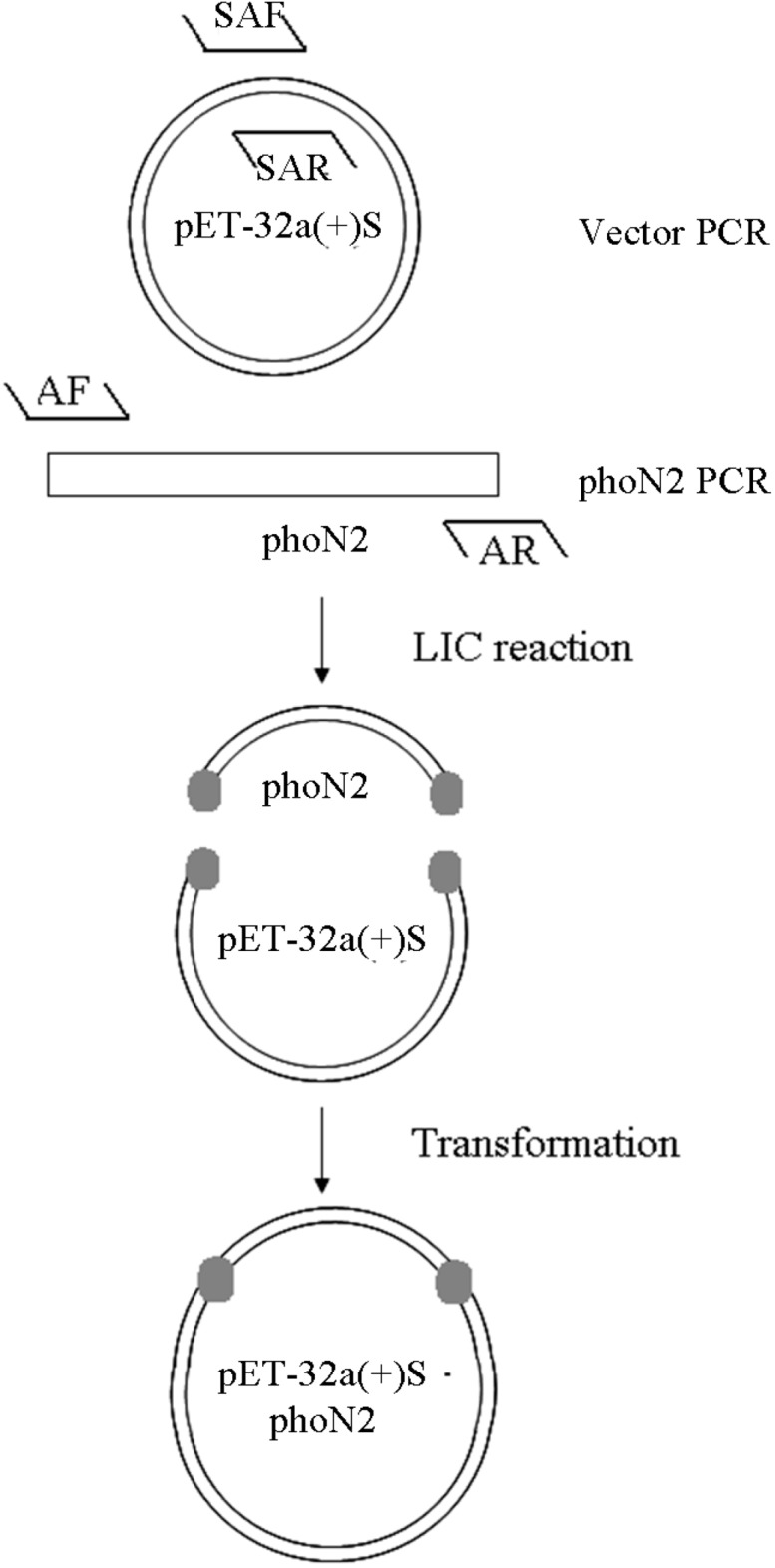
Construction of pET32a (+) S-Apyrase.

### 3.4. Recombinant Protein Expression

The recombinant pET32a (+) S-Ayrase was transformed into *E. coli* BL21 (DE3) and selected on LB agar (ampicillin) at 37 °C overnight. The bacterial colonies were transferred into LB medium and the bacteria were cultured at 37 °C, 200 rpm overnight. In order to prepare the growth curve of the bacteria, the bacteria were sampled at 1, 2, 3, 4, 5, 6, 7 and 8 h. Then OD_600_ of each sample was measured. To determine the fusion of recombinant proteins, the bacteria were cultured at 37 °C, 200 rpm for 4 h, until OD_600_ was 0.6. Then the bacteria were dissolved in PBS and lysed by sonication. The different samples were tested by SDS-PAGE.

### 3.5. Purification of Proteins

The pS-Apyrase protein was purified using S agarose and insoluble material was removed by centrifugation (12,000 r/min, 15 min, 4 °C). Supernatants were filtered through 0.2 μm sterile Acrodisc^®^ syringe filters with membrane onto S agarose and rotated on a rocking platform at 4 °C for 4 h. Beads were collected by centrifugation (3000 r/min, 5 min, 4 °C) and washed three times in a wash buffer (20 mM Tris-HCl, 150 mM NaCl, 1%Triton × (v/v) − 100). The fusion proteins were stored in S agarose beads at 4 °C.

## 4. Conclusions

We have successfully constructed the expression vector pET32a (+) S and recombinant pET32a (+) S-Apyrase by LIC in just one week. Using LIC to construct vectors not only has a higher success rate, but also takes less time. The new method of construction of vectors and recombinant proteins provides an efficient and cost-effective parallel method and thus is applicable for the construction of different vectors, tags and proteins.
